# Quantification of Women Who Could Benefit from Hormone Therapy after Endometrial Cancer Treatment: An Analysis of SEER Data

**DOI:** 10.3390/curroncol29120721

**Published:** 2022-11-26

**Authors:** Ambrogio P. Londero, Anjeza Xholli, Serena Bertozzi, Maria Orsaria, Michele Paudice, Laura Mariuzzi, Angelo Cagnacci

**Affiliations:** 1 Department of Neurology, Rehabilitation, Ophthalmology, Genetics, Maternal and Infant Health (DiNOGMI), University of Genoa, 16132 Genoa, Italy; 2 Academic Unit of Obstetrics and Gynecology, IRCCS Ospedale San Martino, 16132 Genoa, Italy; 3 Breast Unit, University Hospital of Udine, 33100 Udine, Italy; 4 Institute of Pathologic Anatomy, DAME, University Hospital of Udine, 33100 Udine, Italy; 5 Anatomic Pathology Unit, Department of Surgical Sciences, and Integrated Diagnostics (DISC), University of Genoa, 16132 Genoa, Italy; 6 Anatomic Pathology Unit, IRCCS Ospedale San Martino, 16132 Genoa, Italy

**Keywords:** endometrial cancer, endometrioid, survival, hormonal therapy, premenopause, early postmenopause

## Abstract

Our primary aim was to estimate the magnitude of stage I endometrial cancer (EC) survivors that could benefit from hormonal therapy (HT). Our secondary aims were to assess EC incidence in women below 50 and below 60 over the years, and analyze the overall survival and any influencing factors. We analyzed the endometrioid EC data from the Surveillance, Epidemiology, and End Results (SEER) program according to women’s age, tumor stage, and grade. We analyzed the proportions of EC survivors below 50 and below 60 years of age and stratified those age groups by race. For age distribution and survival analysis SEER, 18 registries’ research data (2000–2018) were analyzed. We analyzed the SEER 12 registries’ research data (1992–2019) for incidence time trends. Our investigation found a 14% and 40% cumulative prevalence of stage I EC that occurs in women below 50 or 60 years, respectively. EC’s prevalence has progressively risen in recent decades, but cancer-specific mortality remains low. The increasing number of women affected by EC in premenopause or early postmenopause face an 18 years-survival rate of 96.86% and 95.73%, respectively. A significant proportion of low-grade EC survivors can potentially benefit from HT treatment, and this requires awareness of other aspects of their health or quality of life, in addition to cancer treatments.

## 1. Introduction

Endometrial cancer (EC) is one of the top ten most-diagnosed cancers in women, encompassing 417,000 new diagnoses globally in 2020 [1,2,3]. Europe and North America are among the geographic areas with the most elevated EC rates [4].

The typical surgical management of EC comprises total hysterectomy and bilateral salpingo-oophorectomy, leading to hesitation in surgical menopause in pre-menopausal patients. The hazards associated with hormone therapy (HT) use in stage I or II EC survivors were the object of a recent meta-analysis [5]. The probability of EC recurrence was not increased by HT per survival analysis (HR 0.90, CI.95 0.28 to 2.87) [5]. The analyses, when stratified by tumor stage, hormone therapy type, timing and duration, revealed the same pattern of effects. The recurrence incidence related to HT was considerably higher in black American women (HR 7.58, CI.95 1.96 to 29.31) [5].

HT lowers the incidence of fractures at any point on the skeleton, as well as bone loss, menopausal symptoms, and sexual difficulties. When begun within ten years of menopause or before the age of 60, this reduces the risk of cardiovascular disease [6,7,8]. These protections are strengthened when HT is applied to women experiencing early or premature menopause, including those driven on by ovariectomy, in addition to reducing cognitive impairment [6,7,8].

Cardiovascular mortality is the leading cause of death in EC survivors [9,10], and they share the same manifestations and risks as women without this cancer [11,12,13]. In a Japanese cohort that survived EC, HT produced a superior disease-free survival rate compared to ovarian conservation, according to a recent multicentric retrospective cohort [14]. 

This investigation aimed to determine how many women are eligible for EC treatment and could benefit from HT. To accomplish this, we examined the percentages of women with EC under the ages of 50 and 60 and stratified age ranges by race. The secondary goals were to assess EC incidence in women under 50 and under 60 over time, and look at overall survival and its influencing factors.

## 2. Materials and Methods

### 2.1. Design, Setting, Sample, and Data Extraction

The data used in this retrospective study were obtained from the National Cancer Institute’s Surveillance, Epidemiology, and End Results (SEER) program. SEER gathers and shares cancer-related data from population-based registries, comprising roughly 30% of the U.S. population [15]. Incidence data were extracted from SEER 12 Registries Research Data (1992–2019). Meanwhile, detailed survival data were extracted from SEER 18 Registries Research Data (2000–2018). Data from 2000 onward were utilized, as these encompass periods of increasing endometrial cancer rates. The study was conducted according to the Helsinki Declaration and its subsequent revisions. Institutional review board approval and informed consent were not required for this study because all data extracted from the SEER database are publicly available and wholly deidentified. We signed the data-use agreement and obtained authorization from the SEER program to access and use the data. We followed this agreement while working on this study. 

**Figure 1:**
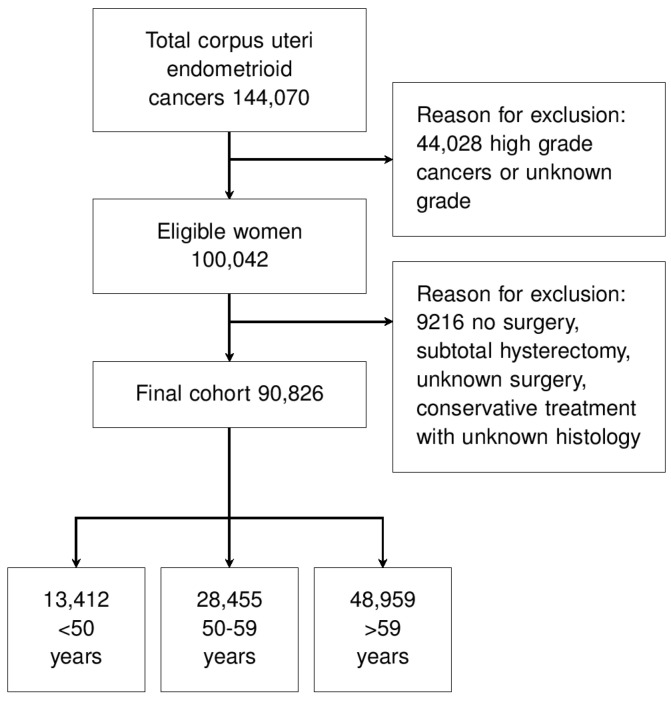
Study flow-chart.

We extracted all cases of corpus uteri endometrioid cancers with tumor grades of G1 or G2. We applied the following exclusion criteria: tumor grading G3 or unknown grading, primary tumor not treated with surgery, subtotal hysterectomy, unknown surgery, and conservative treatment with unknown histology. Patient selection is described in Figure 1.

### 2.2. Data Analysis

We performed all statistical analyses with R software (version 4.2.1) [16]. The p-values were two-sided, and a value below 0.05 was considered statistically significant. The normality of the distribution of continuous variables was tested with Kolmogorov–Smirnov test. Parametric continuous distributions are presented with a mean (±standard deviation) and non-parametric distributions with a median and the interquartile range (IQR). Categorical variables are shown with percentage and absolute values. The missing values were considered missing (NA) in the analyses. Where appropriate, the following tests were used: *t*-test, Wilcoxon test, Fisher’s exact or chi-square test. A false discovery rate adjustment was applied in the multivariate analysis. The hazard ratio (HR) and the overall survival (OS) are presented as their values and a 95% confidence interval (CI.95). The OS was defined as the time from diagnosis to cancer-specific death (only events attributable to the EC). The missing and the unknown causes of death were excluded from the survival analysis. We performed a Kaplan–Meier analysis. We drew the survival curves to assess the OS rates, and the log-rank tests were utilized to investigate the statistical differences between groups. Cox univariate and multivariate regressions were also performed. A sieve bootstrap inference method was used to assess time-trends [17].

## 3. Results

After screening, 90,826 women affected by endometrioid EC were eligible (Figure 1). The reasons for exclusion were high or unknown tumor grade, no surgery for the primary tumor, subtotal hysterectomy, unknown type of surgery, and conservation of the uterus with unknown histology (Figure 1). Table 1 shows the population characteristics. Most cases were tumor stage I and grading G1 (Table 1). 

**Figure 2:**
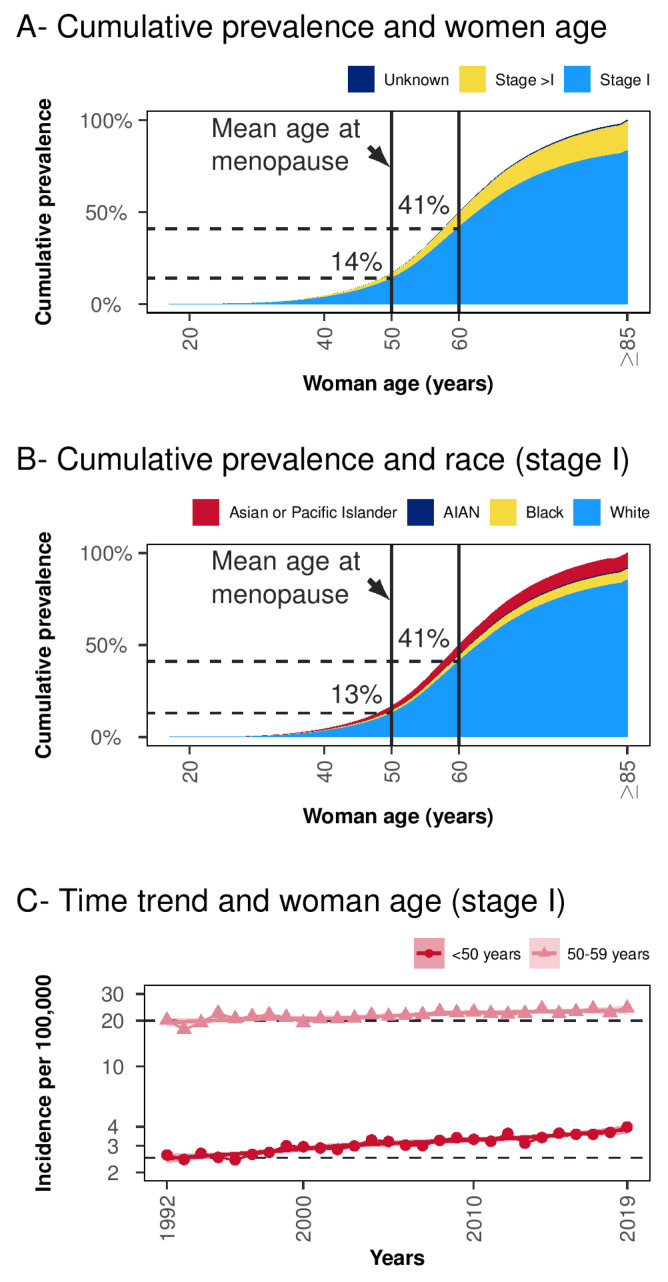
Cumulative women age prevalence and time-trends. Panel (**A**) Cumulative prevalence and women age-stratified per tumor stage. Panel (**B**) Cumulative prevalence and women age in tumor stage I stratified per race (cases with unknown race were excluded). Panel (**C**) Time trend in women below 50 and 50–59 years groups in SEER Research Data (12 Registries, Nov 2021 Sub, 1992–2019). Acronyms: AIAN = American Indian/Alaska Native.

The proportion of women below 50 and below 60 years with stage I EC, was 14% and 41%, respectively (Figure 2A). Similarly, the proportion of white women below 50 and 60 years of age was 13% and 41%, respectively (Figure 2B). According to a sieve bootstrap-based test, a significant linear trend was found for an increasing incidence over the years for stage I EC in women <50 years (*p* < 0.001). The same trend was observed for the age group 50–59 years (*p* = 0.003), although to a lesser extent (Figure 2C). 

**Figure 3:**
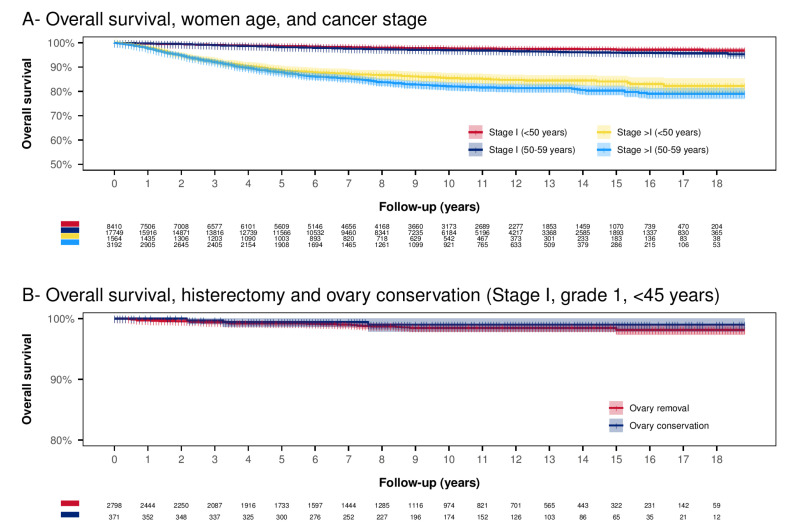
Kaplan–Meier analysis. Panel (**A**) Overall survival according to age category and stage. The Log-rank test results statistically significant for all the paired comparisons. Almost all the differences have *p <* 0.001, except two cases: Stage I (<50 years) vs. Stage I (50–59 years) (*p =* 0.001) and Stage >I (<50 years) vs. Stage >I (50–59 years) (*p =* 0.038). Panel (**B**) Overall survival according to ovary conservation in stage I, tumor grading G1 below 45 years of age (uterus conservation is excluded). The Log-rank test results are not significant (*p =* 0.436).

Considering all stage I EC below 60 years of age, the 18-year OS was 96.11% (CI.95 95.67–96.55%) (Figure 3A). In women <50 years, the 18-year OS was significantly higher than in women 50–59 years of age (96.86%, CI.95 96.06–97.67% vs. 95.73%, CI.95 95.22–96.25%, *p =* 0.001) (Figure 3A). The 18-year OS for EC of stage >in women below 60 years of age, was 80.14% (CI.95 78.34–81.98%). The 18-year OS was higher in women < 50 years of age than in women 50–59 years of age (82.29%, CI.95 79.32–85.37% vs. 79.04%, CI.95 76.83–81.33%, *p =* 0.038) (Figure 3A). Among the low-grade EC, a younger age at diagnosis is a significant protective factor. Moreover, older age at diagnosis, high-grade EC, black race, advanced tumor stage, and G2 grading are substantial predictors for reduced OS (Table 2). 

A possible strategy to remedy the onset of menopause symptoms is to preserve the ovaries at the time of primary surgery. Figure 3B reports survival in women under 45 years, tumor stage I, and grading G1. In this group of women, ovarian conservation shows a non-significant OS improvement (*p =* 0.436). The 10-year OS in the ovarian conservation group was slightly higher than in the group with ovarian removal (respectively 98.99%, CI.95 97.85–100%) vs. 98.45%, CI.95 97.87–99.04%). In multivariate Cox regression, adjusting for possible confounding factors did not lead to any significant difference in OS (HR 0.57, CI.95 0.17–1.89, *p =* 0.359).

## 4. Discussion

### 4.1. Key Results

In SEER data, our analysis found a 14% and 40% cumulative prevalence of stage I EC in women below 50 or 60 years, respectively. This prevalence has been progressively rising in recent decades, but cancer-specific mortality remains low. The increasing number of women affected by EC in premenopause or early postmenopause face an 18-year survival rate, respectively, of 96.86% and 95.73% and require awareness of other aspects related to their health or quality of life. 

### 4.2. Interpretation and Comparison with the Literature

Ovarian conservation may maintain appropriate hormone levels to prevent symptoms of hypoestrogenism. Patients that are eligible for ovarian conservation are limited to women with age equal to or below 45 years, stage I, and low-grade EC with a low risk of ovarian involvement [18]. In a meta-analysis of seven retrospective studies, ovary conservation proved safe and did not increase the recurrence of early stage, low-grade EC [19]. Present data seem to indicate that ovarian conservation in women < 45 years does not affect specific EC mortality. However, ovarian conservation is only used in 8–10% of eligible women with EC [2,14]. The use of HT seems even safer than ovarian conservation on EC risk [18] and would be useful in reducing menopausal symptoms, sexual disturbances, bone loss, fracture risk, cardiovascular risk, and cognitive impairment [6,7,8]. However, no more than 10% EC survivors use HT [18]. 

The main reasons for avoiding HT after surgical removal of stage I low-grade EC are essentially unproven. There is no evidence in vivo that, after surgery, estrogens stimulate the growth of residual microscopic cancer cells [5,20], or that adjuvant therapy targeted to the estrogen pathways, as in breast cancer, has any clinical benefit [21]. Metastatic EC cells has reduced estrogen receptor expression [22]. Cancer progression may depend on alternative oncogenic pathways and not estrogen exposure [20,23]. In addition, high estrogen receptor expression is generally associated with well-differentiated tumors and a favorable prognosis [3,24]. Thus, estrogen may function as a tumor-promoter in orthotopic endometrial tissue without harmful effects after the endometrial tissue’s neoplastic transformation [23]. 

Only black American women revealed a significantly increased risk of recurrence associated with HT (HR 7.58, CI.95 1.96 to 29.31) [5]. Although these findings should be interpreted with caution, because they are derived from a single study, particular attention should be paid to the effects of HT on black American women. They may have some dissimilarities with their white counterpart, such as higher estrogen serum levels and a higher risk of hormone-sensitive benign tumors such as fibroids [25,26]. In addition, as shown in this study, black American women generally have a significantly increased risk of reduced survival.

A previous analysis of the SEER database attributed the racial disparities between the black and white population to the increased advanced-stage, high-grade and aggressive histologic sub-type tumors found in the black population [27]. However, we also found an increased risk of death from cancer in the black population compared to the white population among low-risk EC groups, which could be related to some factors intrinsic to the black population. Among the women affected by breast cancer, the basal-like sub-types, which have an increased risk of adverse outcomes, are more common in young black women than in white ones [26,28,29]. Moreover, despite a similar management and being diagnosed with basal-like breast cancer, black women exhibited a higher risk of nodal recurrence than white women [30]. In addition, basal-like breast cancer in black women has a higher expression of biomarkers associated with aggressive disease [31]. As for breast cancer, while there is a higher prevalence of high-risk basal-like sub-types in young black women than white ones, also in EC, there could be differences in the molecular pattern within the endometrioid histology [26,28,29]. These differences could be partly explained by the difference in the hormone receptors and metabolism observed in the black population [25,26].

### 4.3. Strengths and Weaknesses

The main limitation of the study is its retrospective nature. Another potential limitation of the study is the absence of information about adjuvant treatments, particularly progestogens, in cases of uterus conservation. In addition, SEER 18 represents only around 30% of the US population, potentially limiting generalisability. However, the population nature of the register is also the main strength that allowed for us to assess a large study population (90,826 cases) and have a long-term follow-up (more than 18 years) utilizing high-quality SEER data. This population-based study showed many women who could benefit from HT among EC survivors. The vast population also allowed for us to assess de-escalation management in low-grade EC, such as ovary conservation. 

### 4.4. Relevance of the Results, Unanswered Questions, and Future Research

Overall, the data indicate that about 40% of women surviving early-stage low-grade EC are in the window of opportunity for the HT-eligible population to treat symptoms and prevent long-term consequences of prolonged hypoestrogenism. These findings emphasize the importance of the topic and favor new, adequately powered randomized trials on the benefits and risks of HT use on EC survivors. The only randomized trial published to date was discontinued due to difficulties in enrollment (canceled before reaching the predetermined sample size) [32,33].

## 5. Conclusions

A significant proportion of low-grade EC survivors could potentially benefit from HT treatment; 14% of women are below 50, and 40% are below 60 years of age. The potential candidates for HT are more numerous than the candidates for ovarian preservation. Specific studies are needed to confirm the observational evidence of HT causing no harm to EC recurrence and specific mortality.

## Figures and Tables

**Table 1:** Population characteristics.

Variables	Values
Woman’s age (years)	
<50 years	14.77% (13,412/90,826)
50–59 years	31.33% (28,455/90,826)
>59 years	53.9% (48,959/90,826)
Race	
White	84.75% (76,974/90,826)
Black	5.76% (5228/90,826)
American Indian/Alaska Native	0.67% (607/90,826)
Asian or Pacific Islander	8.2% (7450/90,826)
Unknown	0.62% (567/90,826)
Tumor stage	
Stage I	83.42% (75,764/90,826)
Stage >I	15.76% (14,311/90,826)
Unknown	0.83% (751/90,826)
Tumor grade	
G1	61.73% (56,064/90,826)
G2	38.27% (34,762/90,826)

**Table 2:** Univariate and multivariate Cox regression overall survival analyses.

	HR (CI.95)	*p*	HR (CI.95)(*)	*p*(*)	*p*(†)
Woman’s age (years)					
<50 years	Reference	--	Reference	--	--
50–59 years	1.22 (1.07–1.39)	0.003	1.22 (1.07–1.39)	0.003	0.004
>59 years	2.2 (1.95–2.47)	<0.001	2.1 (1.87–2.37)	<0.001	<0.001
Race					
White	Reference	--	Reference	--	--
Black	1.56 (1.39–1.75)	<0.001	1.51 (1.34–1.69)	<0.001	<0.001
American Indian/Alaska Native	1.22 (0.81–1.85)	0.333	1.54 (1.02–2.33)	0.038	0.049
Asian or Pacific Islander	0.81 (0.71–0.93)	0.003	0.9 (0.78–1.03)	0.132	0.132
Unknown	0.36 (0.15–0.86)	0.022	0.45 (0.19–1.08)	0.072	0.081
Tumor stage					
Stage I	Reference	--	Reference	--	--
Stage >I	5.34 (4.99–5.72)	<0.001	4.53 (4.22–4.86)	<0.001	<0.001
Unknown	2.04 (1.39–2.98)	<0.001	2.06 (1.41–3.01)	<0.001	<0.001
Tumor grade					
G1	Reference	--	Reference	--	--
G2	3.05 (2.84–3.28)	<0.001	2.22 (2.06–2.39)	<0.001	<0.001

(*) Multivariate Cox regression. (†) False discovery rate correction.

## Data Availability

The datasets analyzed during the current study are available in the Surveillance, Epidemiology, and End Results (SEER) database (https://seer.cancer.gov/data/) (accessed on 11 August 2022).
